# Content and trend analysis of user-generated nicotine sickness tweets: A retrospective infoveillance study

**DOI:** 10.18332/tid/145941

**Published:** 2022-03-15

**Authors:** Vidya Purushothaman, Tiana J. McMann, Zhuoran Li, Raphael E. Cuomo, Tim K. Mackey

**Affiliations:** 1 Global Health Policy and Data Institute, San Diego, United States; 2 Division of Infectious Diseases and Global Public Health, School of Medicine, University of California, San Diego, San Diego, United States; 3 Global Health Program, Department of Anthropology, University of California, San Diego, San Diego, United States; 4 S-3 Research, San Diego, United States

**Keywords:** nicotine sickness, ‘nic sick’, vaping, tobacco, Twitter

## Abstract

**INTRODUCTION:**

Exposure to pro-tobacco and electronic nicotine delivery system (ENDS) social media content can lead to overconsumption, increasing the likelihood of nicotine poisoning. This study aims to examine trends and characteristics of nicotine sickness content on Twitter between 2018–2020.

**METHODS:**

Tweets were collected retrospectively from the Twitter Academic Research Application Programming Interface (API) stream filtered for keywords: ‘nic sick’, ‘nicsick’, ‘vape sick’, ‘vapesick’ between 2018–2020. Collected tweets were manually annotated to identify suspected user-generated reports of nicotine sickness and related themes using an inductive coding approach. The Augmented Dickey-Fuller (ADF) test was used to assess stationarity in the monthly variation of the volume of tweets between 2018–2020.

**RESULTS:**

A total of 5651 tweets contained nicotine sickness-related keywords and 18.29% (n=1034) tweets reported one or more suspected nicotine sickness symptoms of varied severity. These tweets were also grouped into five related categories including firsthand and secondhand reports of symptoms, intentional overconsumption of nicotine products, users expressing intention to quit after ‘nic sick’ symptoms, mention of nicotine product type/brand name that they consumed while ‘nic sick’, and users discussing symptoms associated with nicotine withdrawal following cessation attempts. The volume of tweets reporting suspected nicotine sickness appeared to increase throughout the study period, except between February and April 2020. Stationarity in the volume of ‘nicsick’ tweets between 2018–2020 was not statistically significant (ADF= -0.32, p=0.98) indicating a change in the volume of tweets.

**CONCLUSIONS:**

Results point to the need for alternative forms of adverse event surveillance and reporting, to appropriately capture the growing health burden of vaping. Infoveillance approaches on social media platforms can help to assess the volume and characteristics of user-generated content discussing suspected nicotine poisoning, which may not be reported to poison control centers. Increasing volume of user-reported nicotine sickness and intentional overconsumption of nicotine in twitter posts represent a concerning trend associated with ENDS-related adverse events and poisoning.

## INTRODUCTION

The increased consumption of various nicotine products, including electronic nicotine delivery systems (ENDS) has led to an increase in nicotine product-related adverse events^1,2^. Common adverse events range from cough, headaches, throat irritation, nausea, to anxiety, depression, and insomnia^3^. However, severe adverse events are also possible, such as hypertension, tremors, seizures, muscle paralysis, and coma^4,5^. The growing public safety dangers of ENDS were also made evident by the 2019 Outbreak of Lung Injury Associated with e-cigarette use, or vaping (EVALI), which led to a total of 2807 hospitalized cases or deaths in the United States^6^. While acute events such as EVALI have been reported, more mild occurrences may be largely underreported, yet still constituting a significant public safety issue.

ENDS product use has widely expanded with a historical surge in uptake and use among adolescents and young adults^7^. Consequently, the past decade has been characterized as a ‘youth vaping epidemic’, influenced by aggressive marketing campaigns, introduction of various nicotine flavored products, low perception of harm, ability to titrate for higher doses of nicotine, and appeal of product features and convenience, resulting in ENDS now being the most consumed tobacco product among this demographic group^8^. In 2018, 3.05 million US high school and over half a million middle school students reported using ENDS in the past 30 days, representing a 78% and 48% increase from 2017, respectively^9^. Between 2014 and 2020, there was a 122% increase in total ENDS product sales, with JUUL, a ‘pod-mod’ device using nicotine salts, dominating the e-cigarette market^10^.

Increasing social media engagement on ENDS-related topics and challenges can lead to a digital risk environment that promotes use and overconsumption for purposes of gaining online popularity, attention, and social media followers, particularly among adolescents and young adults^7,11^. Specifically, social media vape challenges (e.g. vaping cloud contests) often use hashtags to promote image and video content to other users, which can expose a broader group of social media users to overconsumption behavior, particularly involving high-content nicotine vape products^12^. High nicotine concentration can be found in two major ENDS brands popular among young adults: JUUL and PuffBar, both of which have products which have previously been regarded as unsafe for use^13^. The ease of use associated with these products contributes to increased health risks, with many users not understanding how quickly it is possible to reach excessive nicotine consumption levels. The U.S. Food and Drug Administration (FDA) issued a warning letter on the issue in September 2019^14^.

The growing influence of social media on nicotine use behavior also extends to adverse events attributed to nicotine consumption from ENDS products, where social media user communities have adopted a term popularly referred to as ‘nic sick’, which is short for nicotine sickness, and used to describe non-specific adverse symptoms that occur following exposure to nicotine, especially those attributed to consumption of nicotine above an individual’s tolerance^15^. This term has become an increasingly popular hashtag on social media platforms and can be used to curate user-generated content that details experiences with nicotine sickness, poisoning, and adverse events.

Relatedly, nicotine sickness cases that are not reported to poison control centers have the potential to be identified through alternative surveillance methods, including infoveillance approaches using social media, as has been widely used in other areas of tobacco control research^16–19^. The popular global microblogging platform Twitter is a common infoveillance data source, where 32% of adolescents and 42% of young adults, respectively, report use^20^. Prior studies have used social media to detect adverse events and side effects associated with pharmaceutical products, other medical products, and issues related to substance use disorders^21–24^, with other studies also examining user attitudes and perceptions related to ENDS products, ENDS use behavior, the impact of ENDS product marketing, and even characterizing general adverse effects associated with ENDS^25–28^.

Adding to this body of literature, this study specifically examines the use of ‘nic sick’ on Twitter to identify user-generated content characterizing experiences with nicotine sickness or nicotine poisoning, while also examining trends in the volume of these tweets between 2018 and 2020. It is the first study, to our knowledge, to use the specific term ‘nic sick’ to identify and analyze user-generated discussions on experiencing nicotine sickness on a social media platform. The results of this study aim to provide additional information regarding the potential harms associated with vaping, inform tobacco regulatory science related to ENDS product safety, and ultimately providing insights into characteristics of how online users are experiencing and discussing ENDS-related adverse events that may not be captured in traditional public health surveillance.

## METHODS

This retrospective infoveillance study was conducted in two phases: 1) data collection using the Twitter Academic Research Application Programming Interface (API) stream filtered for ‘nic sick’ specific keywords of interest; and 2) data cleaning, content analysis, and statistical analysis for longitudinal trends.

### Data collection

Tweets related to nicotine sickness were collected from the Twitter Academic Research API using the keywords ‘nic sick’, ‘nicsick’, ‘vape sick’, and ‘vapesick’ between January 2018 and December 2020. The Twitter Academic Research API is a product track that includes access to all API v2 endpoints to help academic researchers use Twitter data. It enables collection of retrospective Twitter data filtered for specific keywords inputted by the user. Search keywords were selected based on relevance to the study objective, related Google Trends search terms for ‘nic sick’, and also determined based on conducing manual searches on Twitter for keywords, terms, and hashtags that were used in conjunction with the primary term ‘#nicsick’. The data collection time frame was based on an analysis of Google Trends data that depicted an increasing trend in search interest for the term ‘nic sick’ during this period^29^. Retweets were removed and the collected tweets with underlying metadata (date and time at which the tweet was created, hyperlink to the tweet, text) were exported to a password-protected database for manual content coding and further statistical analysis.

### Data analysis

*Content coding*

Manual annotation of the collected tweets was conducted by authors VP and TJM. Inductive content analysis of the collected tweets included a coding scheme for binary classification of whether the tweet discussed content related to nicotine poisoning or nicotine-related adverse events (e.g. vomiting, nausea, headache, burning sensation in throat, fatigue of varied severity). Specifically, user-generated tweets with content related to: 1) adverse effects during or after nicotine sickness; and/or 2) users soliciting comments or suggestions to overcome nicotine sickness symptoms were labelled as ‘signal’ tweets (i.e. tweets relevant to the study aims). The authors also denoted whether the tweet reflected firsthand or secondhand experiences related to nicotine sickness or other adverse events following nicotine consumption. We also excluded tweets that did not appear to originate from individual twitter users (e.g. organizational accounts, ads, bots, etc.) and that included content specific to news about ENDS adverse events and health effects, public service announcements about harms of ENDS, and marketing or promotion of ENDS products, hereinafter referred to as ‘noise’. The coding scheme was further expanded to include emerging subthemes for signal tweets related to nicotine sickness using an inductive coding approach (see Supplementary file for description of coding scheme). The authors also annotated tweets that discussed adverse effects or symptoms associated with nicotine withdrawal. VP and TJM coded the posts independently and achieved a high intercoder reliability score (kappa=0.95) for signal classification and major themes coded. For inconsistent results, authors reviewed and conferred on the correct classification with authors RC and TKM.

### Statistical analysis

The volume of signal tweets with content related to nicotine sickness was stratified by month, as well as by quarter, from January 2018 to December 2020, permitting the calculation of descriptive statistics and further statistical analysis. The Augmented Dickey-Fuller (ADF) test was used to assess the statistical significance of the monthly variations in the volume of signal tweets related to nicotine sickness from January 2018 to December 2020. The ADF test assesses for a unit root (a characteristic of a time series that renders it non-stationary) including a higher regressive order process in the model. While the alternative hypothesis for the ADF test represents stationarity or a constant trend, the null hypothesis represents non-stationarity in the time series for the volume of signal tweets related to nicotine sickness. A p-value of 0.05 was considered the significance threshold level and a p-value of <0.05 was considered to indicate that the time series was stationary. All statistical analyses were conducted in RStudio version 3.6.1 using the time series analysis package (‘tseries’).

## RESULTS

A total of 5651 tweets with the keywords ‘nic sick’, ‘nicsick’, ‘vape sick’, and ‘vapesick’ were collected between January 2018 and December 2020. After manual annotation using our inductive coding approach, 18.3% (n=1034) of the tweets were confirmed as including user-generated content discussing nicotine sickness (Supplementary file). Noise we detected in this dataset included tweets posting the meaning of ‘nicsick’ or tweets unrelated to nicotine sickness. After inductive coding, signal tweets with user-generated content related to nicotine sickness or nicotine poisoning were first categorized and reported as either: 1) firsthand reporting; and 2) secondhand reporting of suspected nicotine sickness symptoms (e.g. vomiting, nausea, headache, burning sensation in throat, fatigue of varied severity). Nearly 9 in 10 signal tweets (89.6%) discussed self-reported experiences of specific ‘nic sick’ symptoms and just over 10% of the signal tweets (n=108) reported observations of other users experiencing nicotine sickness (see Table 1 for de-identified and paraphrased example tweets).

**Table 1:** Volume and examples of tweets related to nicotine sickness symptoms (text paraphrased for deidentification)

*Themes/subthemes*	*Tweets^a^ n (%)*	*Examples^b^*
**Self-reporting of symptoms** (firsthand)	926 (89.56)	‘Did I get myself nic sick again purely because I am sad and angry? yes. will I do it again? Yes.’ ‘I' l just hit some nic and I have not hit it in this manner in like 3 months and now I so sick oh my god.’
**Secondhand reporting of symptoms**	108 (10.44)	‘I was leaving the bathroom when I saw three girls vomiting from being nic sick: The vibes were not right for you today girls.’
Intentional overconsumption of nicotine	8 (0.01)	‘You can laugh all you want but I will be over here enjoying my sick nic buzz.’
Intent to quit	13 (0.01)	‘The only good thing that happened in 2019 was I quit vaping during summer because I got nic sick and vomited on the side of the road and I think it is disgusting.’
Mention of product type/brand	193 (18.67)	‘Purchased juul and getting nic sick very often, although I am not even vaping it continuously, strange. Is anyone else facing same issues?’ ‘I got nic sick then felt better then used my bootleg puff bar and now I am nic sick again.’
Withdrawal (not related to nicotine sickness)	9 (0.01)	‘@XXXXX cold turkey helps to cut it off, if that does not work for you then try reducing the nic percent following which you will feel sick for some time and will not feel good but you will feel so much better without it when the withdrawals are over.’

a Number of tweets and the percentage of total signal tweets that contained the theme.

b Tweet text paraphrased for de-identification.

Signal tweets were also grouped into four sub-thematic categories based on inductive coding: 1) intentional overconsumption of nicotine products to ‘get high’ or ‘feel nic buzz’ in spite of being aware of the consequent adverse effects; 2) users expressing intention to quit after experiencing ‘nic sick’ symptoms; 3) users mentioning the nicotine product type or brand name that they consumed in the same tweet expressing nicotine sickness related adverse effects; and 4) users discussing symptoms associated with nicotine withdrawal following cessation attempts (see Table 1 for example tweets in each category).

While only a minority of the signal tweets discussed intentional overuse (0.01%), intent to quit (0.01%), and withdrawal symptoms (0.01%), 81.33% (n=841) included users sharing specific nicotine sickness experiences without specifically mentioning the ENDS product or brand name. However, nearly one-fifth of the signal tweets (n=193; 18.67%) discussed both experiences with specific nicotine sickness symptoms and included users mentioning the product type and/or brand associated with use. Among the signal tweets that mentioned brand names (n=81), over 3 in 5 (n=51; 62.9%) mentioned JUUL and nearly one-quarter (n=19; 23.5%) mentioned Puff Bars. Among the signal tweets that mentioned product type without brand names (n=112), over half mentioned experiencing nicotine sickness symptoms during or after vaping (n=62; 55.4%) and nearly one-fifth mentioned cigarettes (n=22; 19.64%). Other product types and brand names mentioned included NJOY, salt ‘nic vapes’, Newport (combustible), and hookah.

**Figure 1:**
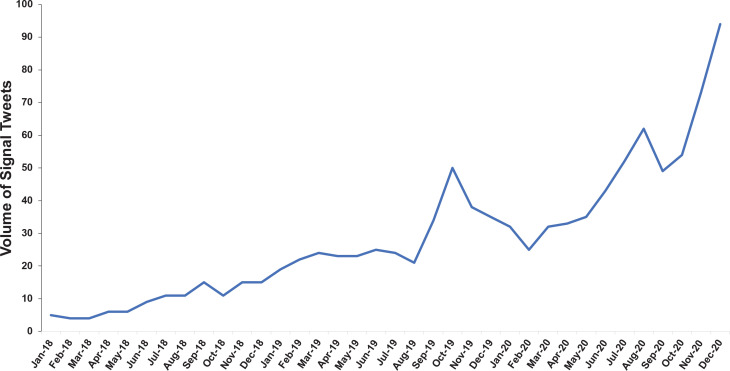
Volume of signal tweets related to nicotine symptoms from January 2018 to December 2020

Following content analysis, the volume of all signal tweets was then stratified by quarter and month from January 2018 to December 2020. The final quarter of the sample (Oct – Dec 2020) had the highest volume of signal tweets related to nicotine sickness (n=221; 21.4%), followed by the penultimate quarter of the sample (Jul – Sep 2020; n=163; 15.8%), and fourth quarter of year 2019 (Oct – Dec 2019; n=123; 11.9%). The monthly volume of signal tweets related to nicotine sickness was second highest in November 2020 (n=73; 7.1%) and highest in December 2020 (n=94; 9.1%), both dates occurring after peaks in EVALI cases in September 2019. However, the highest percentage increase in the monthly volume of signal tweets was observed from August 2019 to September 2019 (62.9% increase), a time when EVALI cases were rapidly increasing. Overall, the volume of signal tweets related to nicotine symptoms showed an increasing trend from January 2018 to December 2020 (Figure 1).

A time series was created for the volume of monthly signal tweets to assess its stationarity. The Augmented Dickey-Fuller (ADF) test on the time series of volume of signal tweets related to nicotine sickness indicated a non-stationary change in the volume of signal tweets (ADF = - 0.32, p=0.98). This non-stationary monthly time series in the volume of tweets related to nicotine sickness suggests an increasing trend in user-generated ‘nic sick’ conversations on Twitter during the study period.

## DISCUSSION

Our study found that user-generated content related to nicotine sickness keywords and hashtags on Twitter includes open discussion of user experiences with varied adverse effects following nicotine consumption, with the overall volume of these posts increasing over time. This study also captured both firsthand and secondhand reporting of nicotine sickness symptoms, with most of these tweets discussing self-reported experiences of more minor symptoms of nicotine sickness and/or seeking help from online communities to overcome the adverse effects.

Additionally, among the signal tweets with product brand names, the majority mentioned JUUL and PuffBar, which are two of the usual brands of e-cigarettes used among middle and high school students^30^. EVALI outbreak data published by the U.S. Centers for Disease Control and Prevention (CDC) showed that the number of EVALI cases peaked during the month of September 2019 following a sharp increase in August 2019^31^. Similarly, our study observed the highest increase in tweets related to nicotine sickness from August 2019 to September 2019 (62.9% increase), though the majority of these tweets did not mention symptoms or cases that exhibited EVALI-related complications and none mentioned products containing THC or Vitamin E acetate, both substances implicated in the EVALI outbreak. Specifically, our study considered nicotine sickness following exposure to any nicotine-containing product, including combustibles, e-cigarettes, and other products, though the vast majority of tweets related to ENDS adverse events.

The American Association of Poison Control Centers publishes monthly data on the prevalence of cases resulting from exposure to e-cigarettes and liquid nicotine^32^. These data include closed human exposures to e-cigarettes and liquid nicotine reported to poison centers. Evidencing increased risks, nicotine exposure cases reported to the National Poison Data System increased nearly 5000% from 2010 to 2018, with over 10800 cases alone in 2019^33^. Data from this study bear a similar overall increase to the number of exposure cases observed in 2018 and 2019, with the highest number of monthly exposure cases reported in September 2019 (871 exposures), coinciding with the sharp increase in the volume of signal tweets (62.9% increase) related to nicotine sickness that we observed on Twitter. However, the trend in monthly number of exposure cases from e-cigarettes and liquid nicotine follows a different pattern to the trend in the volume of signal tweets observed in 2020. Poison control data showed an almost steady number of exposure cases through 2020, with the highest number reported in January 2020 (371 exposure cases) and lowest in March and April 2020 (270 exposure cases each month). However, this study observed an increasing trend in the volume of tweets related to nicotine sickness in the year 2020, with the highest volume of tweets reported during December 2020.

Discrepancies between poison center data and user-generated comments on a single social media platform may be attributed to a number of different factors such as the COVID-19 pandemic, which may have reduced the number of poisonings that were being reported to poison control centers. Also, cigarette sales in 2020 increased for the first time in two decades according to the Federal Trade Commission annual cigarette report, which may also have influenced adverse event attributed to ENDS^34^. Differences may also reflect different modalities in how people report nicotine-related adverse experiences, with milder symptoms that do not involve intervention or hospitalization more likely to be reported through user-generated comments on the Internet versus a formal report to the poison control centers. Hence, these results further emphasize the need for more robust and multimodal forms of active surveillance for nicotine and ENDS-related adverse events that combine structured sources (e.g. surveys, poison control data, FDA adverse event data) and unstructured sources (e.g. social media posts, web forums, Internet search trends) to allow a more complete capture of nicotine poisoning prevalence.

In fact, milder nicotine sickness symptoms may be underreported due to the inaccessibility or lack of convenience of traditional adverse event reporting systems (e.g. FDA Adverse Event Reporting System reports primarily originate from clinicians and manufacturers, not end-users), leading users to report these instances on more accessible and social-driven platforms such as Twitter^19,35^. For example, a prior study on tweets about acute nicotine toxicity due to intentional or accidental exposure to e-liquid in ENDS reported that over 60% of the exposure tweets between 2013–2018 were classified as accidental exposure^36^. Our results may also provide insights that are ENDS product specific, with the potential to generate regulatory approaches that are risk-based and sensitive to the introduction of new nicotine products where the short-term and long-term health impacts are less known^37^. Finally, <1% of posts we reviewed included discussion about users intending to quit, even though users in these groups actively discussed the clear health risks and adverse outcomes associated with these addictive products. Hence, findings from this and other infoveillance studies can aid in formulating targeted anti-tobacco content for the purposes of increasing awareness of nicotine-related adverse effects and possibly facilitating cessation of nicotine product use. Health promotion and education that focuses on digital audiences should consider these findings as an opportunity to actively enter the ‘nic sick’ discussion, and hopefully generate more opportunities to reduce nicotine-related harms.

### Limitations

Data for this study were collected from a single social media platform, Twitter, which may not be representative of all user-generated content related to nicotine sickness on social media, especially as user characteristics may vary from one social media platform to another. Hence, future studies should conduct cross-platform infoveillance studies for nicsick-related terms and assess how users discuss these topics differently depending on the platform used and the ways it encourages user interaction and information sharing. Additionally, the keywords used in the study were chosen based on manual searches on the platform and an initial analysis of Google Trends data, but may not have captured all keywords or hashtags for relevant conversations related to the study aims. For example, there may be other hashtags with similar descriptive terms (e.g. #thenicksicknetwork, #nicotinepoisoning) on Twitter or other social media platforms that may include relevant and non-relevant conversations related to nicotine poisoning that require further analysis. Further, user characteristics of Twitter accounts may not reflect nicotine users in the general population, thereby limiting generalizability. For example, although we compared study results with publicly available reports from the American Association of Poison Control Centers, user-generated reporting could not be cross-validated with other data sources. Also, data collection was not geographically bound to restrict posts from users outside the United States. Furthermore, while the ADF statistical test indicated significant non-stationarity in the monthly volume of signal tweets, this test alone does not indicate any seasonality and how seasonality could affect volume. Also, the number of reported nicotine sickness discussions on social media platforms likely only constitutes a proportion of the true incidence and underestimates the morbidity burden due to nicotine sickness, as many users may not participate or comment via social media or specifically on Twitter.

## CONCLUSIONS

Results point to the need for alternative forms of adverse event surveillance to appropriately capture the growing health burden of vaping. Infoveillance approaches on social media platforms can help to assess the characteristics of user-generated content discussing suspected nicotine poisoning, which may not be reported to poison control centers. Increasing twitter activity on nicotine sickness and intentional overconsumption represents a concerning trend further highlighting the potential underreported health harms of ENDS.

## Data Availability

The data supporting this research are available from the authors on reasonable request.
