# Supplementary material for: Content and trend analysis of user-generated nicotine sickness tweets: A retrospective infoveillance study
Source: Tob Induc Dis. 2022 Mar 15;20:30. doi: 10.18332/tid/145941 (PMC8919180; doi:10.18332/tid/145941)
Supplement: Supplementary file 1 [file TID-20-30-s1.pdf]

Supplemental file 1: Coding Scheme for Tweets

Manual annotation of Tweets: The following coding scheme was used for binary classification of whether the tweet discussed nicotine sickness (including first-hand or second-hand experiences), intentional overconsumption of nicotine, intent to quit, mention of product type/brand, or withdrawal symptoms not related to nicotine sickness.

THEME/SUB-THEME	SIGNAL	CODING SCHEME
**Nicotine Sickness Symptom Reporting** (First-hand/Second-hand)	YES	• Mention of specific nicotine sickness symptoms such as vomiting, nausea, headache, burning sensation in throat, fatigue (varied severity) during or after nicotine use.<br>• Reporting nicotine sickness symptoms of self or friends/family/neighbors (mention of relationship, tagging the profile of other users).<br>• Reporting visiting clinic/urgent care/ER or hospitalization for alleged nicotine sickness symptoms.<br>• Users seeking suggestions on overcoming nicotine sickness symptoms.
	NO	• Tweeting news posts related to nicotine sickness.<br>• Tweets on nicotine sickness symptoms by users or public health organizations to create awareness about nicotine sickness.<br>• Nicotine sickness due to exposure in tobacco fields.<br>• Tweets unrelated to nicotine sickness using the hashtag “nic sick”.<br>• Sarcasm/jokes about nicotine sickness.
**a. Intentional overconsumption of nicotine**	YES	• Reporting intentional use of nicotine over and above one’s tolerance level to experience “nicbuzz”.

**b. Intent to quit**	YES	• Users discussing nicotine sickness symptoms and an alleged intent to quit nicotine use due to unpleasant adverse effect.
**c. Mention of product type/brand**	YES	• Users discussing nicotine sickness symptoms during or after use of a specific nicotine product or brand (e.g., JUUL, Puff Bar etc.,)
**d. Withdrawal symptoms (not related to nicotine sickness)**	YES	• Users discussing varied symptoms or adverse effects associated with nicotine withdrawal.

© 2022 Purushothaman V. et al.
